# Coronary Artery Disease with Elevated Levels of HDL Cholesterol Is Associated with Distinct Lipid Signatures

**DOI:** 10.3390/metabo13060695

**Published:** 2023-05-26

**Authors:** Wanying Xia, Haiyi Yu, Guisong Wang

**Affiliations:** Department of Cardiology and Institute of Vascular Medicine, Peking University Third Hospital, NHC Key Laboratory of Cardiovascular Molecular Biology and Regulatory Peptides, Key Laboratory of Molecular Cardiovascular Science, Ministry of Education, Beijing Key Laboratory of Cardiovascular Receptors Research, No. 49 North Garden Road, Haidian District, Beijing 100191, China; wanyingxia95@gmail.com

**Keywords:** lipidomics, coronary artery disease, high-density lipoprotein cholesterol, sphingolipid, glycerophospholipid, biomarker

## Abstract

Levels of high-density lipoprotein cholesterol (HDL-C) are inversely associated with the incidence of coronary artery disease (CAD). However, the underlying mechanism of CAD in the context of elevated HDL-C levels is unclear. Our study aimed to explore the lipid signatures in patients with CAD and elevated HDL-C levels and to identify potential diagnostic biomarkers for these conditions. We measured the plasma lipidomes of forty participants with elevated HDL-C levels (men with >50 mg/dL and women with >60 mg/dL), with or without CAD, using liquid chromatography–tandem mass spectrometry. We analyzed four hundred fifty-eight lipid species and identified an altered lipidomic profile in subjects with CAD and high HDL-C levels. In addition, we identified eighteen distinct lipid species, including eight sphingolipids and ten glycerophospholipids; all of these, except sphingosine-1-phosphate (d20:1), were higher in the CAD group. Pathways for sphingolipid and glycerophospholipid metabolism were the most significantly altered. Moreover, our data led to a diagnostic model with an area under the curve of 0.935, in which monosialo-dihexosyl ganglioside (GM3) (d18:1/22:0), GM3 (d18:0/22:0), and phosphatidylserine (38:4) were combined. We found that a characteristic lipidome signature is associated with CAD in individuals with elevated HDL-C levels. Additionally, the disorders of sphingolipid as well as glycerophospholipid metabolism may underlie CAD.

## 1. Introduction

Globally, coronary artery disease (CAD) is the leading cause of death and a major public health concern [1]. The pathological basis of CAD is atherosclerosis, which could be due to several risk factors, such as dyslipidemia, hypertension, and smoking [2]. As a significant component of lipids, high-density lipoprotein cholesterol (HDL-C) has been proven by epidemiological studies to have an inverse association with CAD [3]. However, numerous drugs designed to enhance HDL-C levels failed to confer significant benefits toward the reduction in cardiovascular disease (CVD) events and death risk [4,5]. Moreover, a significant number of people suffer from coronary events despite having favorable HDL-C levels, and some patients with CAD have even been reported to have high HDL-C levels [6]. An analysis of two prospective studies proves a higher HDL-C level is a significant major cardiac event risk factor [7]. Additionally, elevated plasma levels of HDL-C caused by several genetic variants are found to be related to an increased risk of ischemic heart disease [8,9,10,11]. The mechanism underlying the occurrence of CAD in patients with elevated HDL-C levels is not fully elucidated. In addition to the concentration, the biological dysfunction of HDL itself can influence the effect of this lipid on CAD [12]. However, our previous study, the Peking University Health Science Center and University of Michigan Medical School Study of Cardiovascular Diseases (PUHSC-CVD), found no significant differences in reverse cholesterol transport (RCT), which is the prominent function of HDL, between patients with CAD and controls with concurrently increased HDL-C concentrations [13]. This finding has been replicated by another study [14]. Besides HDL itself, changes in plasma composition accompanied by increased HDL-C, which might contribute to the onset of CAD, remain to be discovered.

Long-term clinical studies [2,3,4] show that lipids play a prominent role in CAD pathogenesis and progression. To study this, recent developments in lipidomics have led to the design of powerful tools that can deepen our understanding of diseases with lipid dysfunction, as well as aid in the discovery of putative lipid biomarkers, through application of such approaches to study lipidomes using analytical chemistry. Such studies have revealed the presence of abnormal lipidomes in CAD and atherosclerosis [15,16,17,18,19,20]. Observational studies identified altered signatures in lipid metabolism of patients with angina or myocardial infarction [16,17]. Several studies have demonstrated that perturbed sphingolipid metabolism and glycerophospholipid metabolism are factors contributing to CAD [18,19]. Furthermore, studies found that the lipid components of HDL in patients with CAD are different from those in controls [21,22]. Additional investigations have suggested that altered lipid profiles detected in CAD subjects could be informative as diagnostic and prognostic biomarkers [23]. Data from large clinical studies demonstrated that a ceramide- and phospholipid-based risk score was shown to be an efficient predictor of residual CVD event risk in patients with CAD after validation [24]. A new model combining six lipids and traditional factors predicts adverse cardiovascular events in patients with coronary total occlusion after percutaneous coronary intervention effectively [25]. Based on these findings, we hypothesize that plasma lipidome of individuals with increased HDL-C levels plays an important role in CAD incidence and has the potential to be diagnostic markers, which have not been reported to date.

In this study, we performed a plasma lipidomic evaluation in individuals with high HDL-C levels using the liquid chromatography–tandem mass spectrometry (LC-MS/MS) method. The lipidome of twenty patients with CAD was compared to that of twenty controls without CAD to assess the distinction between both groups and to identify CAD-related lipids. We further subjected selected metabolites to pathway analysis to explore how lipid dysregulation contributes to CAD pathophysiology. The diagnostic potency of differential lipids was also calculated to evaluate their utility as lipid biomarkers for CAD.

## 2. Materials and Methods

### 2.1. Participants

We recruited 40 participants with high HDL-C levels (men with HDL levels > 50 mg/dL and women with HDL levels > 60 mg/dL) from the PUHSC-CVD cohort. As previously reported [13], patients meeting the following criteria were included: subjects between 30–75 years old; in those with CAD, coronary stenosis ≥ 50% confirmed in at least one of the main branches of the coronary arteries as indicated by coronary angiography (CAG) and/or cardiac computed tomography (CT) angiography; in those without CAD, normal coronary arteries or only irregularities or coronary stenosis ≤ 20% in the main branches of the coronary arteries as confirmed by CAG and/or CT. The exclusion criteria were as follows: subjects with acute myocardial ischemia, history of previous myocardial infarction, history of diabetes mellitus, with left ventricular ejection fraction < 30% or end-stage congestive heart failure (New York Heart Association functional class III or IV), renal dialysis or severe chronic kidney disease, active liver disease or hepatic dysfunction, use of statins within the past 3 months, and pregnant or lactating women. More details about diagnosis of complications can be referred to our previous work [13].

Based on CAG or CT, all participants were divided into two groups (20 patients per group): the high HDL-C with CAD (High HDL CAD [+]) group and the high HDL-C without CAD (High HDL CAD [−]) group. This study was conducted according to procedures outlined in the Declaration of Helsinki and its later amendments. All enrolled patients provided written informed consent before participating, and the study was approved by the Ethics Committee of Peking University Health Science Center (ID: IRB00001052-11064).

### 2.2. Sample Preparation, Lipid Extraction, and LC-MS/MS Lipidomic Analysis

#### 2.2.1. Sample Preparation

Peripheral blood samples for laboratory tests were collected from subjects after an overnight fast, on the second day of admission. Clinical data were obtained by using standard laboratory methods at Peking University Third Hospital. Blood samples for lipidomic detection were collected immediately after CAG or CT. Sample processing and storage methods have been thoroughly described in our previous report [13].

#### 2.2.2. Lipid Extraction

Lipid extraction was performed following the improved Bligh and Dyer’s protocol [26,27]. All plasma work-ups were performed at 4 °C. Plasma was obtained by centrifugation at 2000 rpm for 10 min; it was then deactivated with a 750 μL mixture of chloroform/methanol (*v*/*v* 1:2). Samples were incubated for 1 h in a vacuum chamber in a dark room after being vortexed for 15 s. Thereafter, 250 μL of chloroform and 350 μL of deionized water were added to the mixture, and both were ice-cold. The homogenate was vortexed for 15 s, put on ice for 1 min, and centrifuged at 12,000 rpm for 5 min. The bottom organic phase was transferred to a new tube. The remaining aqueous phase was mixed with 450 μL ice-cold chloroform for the second extraction step. After the mixture, the aqueous phase was then centrifuged at 12,000 rpm for 5 min to extract the lower organic phase. After two rounds, the extracts of the organic phase were pooled together and dried in the SpeedVac under OH mode. The dried extracts were stored at −80 °C until further lipidomic analysis.

#### 2.2.3. LC-MS/MS Lipidomic Analysis

Plasma lipid profiles were measured by a high-coverage targeted lipidomics approach using liquid chromatography (LC) multiple reaction monitoring, based on an extensive library tailored for human lipidome by LipidALL Technologies Company Limited (Changzhou, Jiangsu Province, China) [26,28]. All lipidomic detection procedures were performed on an Exion UPLC coupled with a QTRAP 6500 PLUS system (Sciex). MS analyses were performed in the electrospray ionization mode (ESI) mode under the following conditions: curtain gas = 20 psi, ion spray voltage = 5000 V, temperature = 400 °C, ion source gas 1 = 35 psi, and ion source gas 2 = 35 psi. Before analysis, plasma lipid extracts of the organic phase were resuspended in 100 μL of chloroform/methanol (*v*/*v* 1:1) using appropriate internal standards. The lipids from different classes were relatively quantified using their respective internal standards.

Individual lipid classes of polar lipids were separated using a Phenomenex Luna 3 μm-silica column (length × internal diameter: 150 mm × 2.0 mm) under the following chromatographic conditions: mobile phase A (chloroform: methanol: ammonium hydroxide, 89.5:10:0.5) and mobile phase B (chloroform: methanol: ammonium hydroxide: water, 55:39:0.5:5.5). The gradient of phase A began with 95% and was held for 5 min, which was then decreased to 60% of A linearly within 7 min. Thereafter, it was maintained at 60% for 4 min. The gradient was further reduced to 30% A and was kept for 15 min before returning to the initial level. The primary gradient was finally maintained for 5 min. Subsequently, a comparative analysis of various polar lipids was performed by establishing multiple reaction monitoring transitions. Isolated polar lipid species, including d_31_-phosphatidylcholine (PC)-16:0/18:1, d_31_-phosphatidylethanolamine (PE)-16:0/18:1, d_31_-phosphatidylserine (PS)-16:0/18:1, d_31_-phosphatidylinositol (PI)-16:0/18:1, d_31_-phosphatidic acid (PA)-16:0/18:1, PA-17:0/17:0, d_31_-phosphatidylglycerol (PG)-16:0/18:1, lyso-PC (LPC)-17:0, lyso-PE (LPE)-17:1, lyso-PS (LPS)-17:1, lyso-PA (LPA)-17:0, lyso-PI (LPI)-17:1, ceramide (Cer)-d18:1/17:0, glucosylceramide (GlcCer)-d18:1/8:0, d3-lactosylceramide (LacCer)-d18:1/16:0, d3-monosialo-dihexosyl ganglioside (GM3)-d18:1/18:0, sphingosine-1-phosphate (S1P)-d17:1, were quantified according to spiked internal standards. Quantification of glycerol lipids, such as diacylglycerols (DAGs) and triacylglycerols (TAGs), was performed using a modified version of reverse phase HPLC/ESI/MS/MS. Briefly, the aforementioned lipids were separated on a Phenomenex Kinetex-C18 2.6 mm column (length × internal diameter: 100 mm × 4.6 mm) with an isocratic mobile phase of chloroform: methanol: 0.1 M ammonium acetate (*v*/*v*/*v* 100:100:4) at a flow rate of 160 μL/min for 20 min. Based on neutral loss-based MS/MS techniques, concentrations of TAGs were determined using d_5_-TAG (16:0)_3_, d_5_-TAG (14:0)_3_, and d_5_-TAG (18:0)_3_ as internal standards, whereas levels of DAGs were calculated in reference to spiked d_5_-DAG (1,3-16:0) and d_5_-DAG (1,3-18:1). Using the HPLC/atmospheric pressure chemical ionization (APCI)/MS/MS mode, free cholesterols, cholesteryl esters (CE), and free fatty acids were separated using d_6_-cholesterol and d_6_-CE18:0 as internal standards. Details of these materials are provided in Table S1.

### 2.3. Statistical Analysis

We analyzed the clinical data using SPSS (IBM SPSS Statistics for Windows, Version 26.0. Armonk, New York, USA: IBM Corp). A Shapiro–Wilk test was used to test the goodness of fit of continuous variables. Continuous variables are expressed as means ± standard deviation or median (interquartile range), whereas dichotomous variables are expressed as percentages. For continuous variables, the Student’s t-test was used to compare normally distributed variables, while the Mann−Whitney U test was performed in non-normally distributed variables. Fisher’s exact test was applied to dichotomous variables.

After log transformation, concentrations of identified lipids were analyzed by multivariate and univariate statistical methods using MetaboAnalyst 5.0 (http://www.metaboanalyst.ca/, accessed on 23 May 2023). Multivariate analyses, including principal component analysis (PCA) and partial least-squares-discriminant analysis (PLS-DA), were performed to distinguish between patients with CAD and controls. PLS-DA was performed as a supervised approach to recognize important variables with discriminative power by calculating variable importance in projection (VIP) scores. For univariate analysis, statistical significance of features was conducted using the Wilcoxon rank-sum test with a threshold of *p* < 0.05 and fold change (FC). FC was calculated based on the mean ratios for lipids of the High HDL CAD (+) group to lipids of the High HDL CAD (−) group. Adjusted *p* value was calculated using logistic regression adjusting age, sex, smoking status, and hs-CRP (hypersensitive C-reactive protein).

The differential lipid species were determined by the results of multivariate and univariate analyses, shown by a volcano chart generated by GraphPad Prism 8.0.2 (GraphPad Software Inc., La Jolla, CA, USA) and boxplots by R software version 4.2.2 (R Foundation for Statistical Computing). Subsequently, we applied hierarchical clustering analysis (HCA) to selected lipids to observe the overview trend, which was visualized by a heatmap. Pathway analysis was performed to explore potential underlying lipid pathway perturbations. HCA and pathway analyses were performed using MetaboAnalyst 5.0. Additionally, the Spearman correlation analysis was performed using SPSS 26.0. The correlation network between significantly altered lipid metabolites was visualized using Cytoscape 3.7.2, according to Spearman correlation coefficients.

The classical univariate receiver operating characteristic (ROC) analysis was adopted to evaluate the performance of differentially expressed lipids in CAD diagnosis and calculate the area under the curve (AUC) with 95% confidence intervals, sensitivity, and specificity, which were the basis for selecting potential biomarkers. Thereafter, we created a diagnostic model by combining the identified biomarker candidates. The ROC analysis and model creation were conducted by MetaboAnalyst 5.0.

## 3. Results

### 3.1. Characteristics of Study Participants

Clinical and demographic characteristics of the study population are described in Table 1. The enrolled patients did not significantly differ in age, sex, waist circumference, body mass index, smoking status, and history of hypertension. Regarding the data on lipid levels, the High HDL CAD (+) group had higher apolipoprotein B (Apo B) and lipoprotein (a) levels than the CAD (−) group. However, no significant differences in HDL-C, total cholesterol, low-density lipoprotein cholesterol, total triglyceride, and apolipoprotein A1 were observed between the two groups. Moreover, hs-CRP levels were higher in samples collected from the CAD (−) group.

### 3.2. Comparison of Plasma Lipidomic Profiles between the High HDL CAD (+) and High HDL CAD (−) Groups

**Figure 1:**
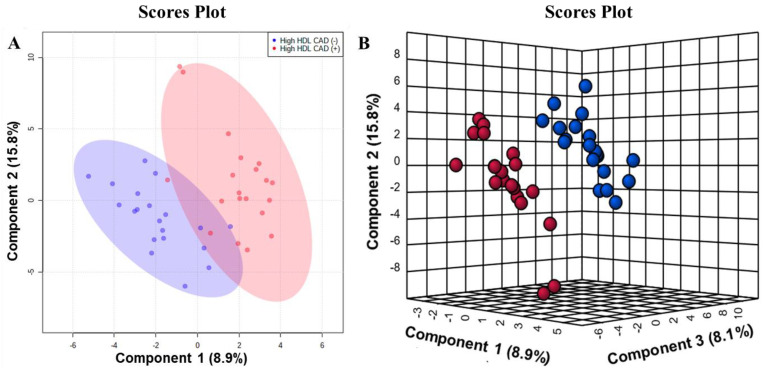
Partial least−squares−discriminant analysis (PLS−DA) scores plot of the High HDL CAD (+) and High HDL CAD (−) groups. (**A**) Two−dimensional scores plot with 95% confidence regions. (**B**) Sparse three−dimensional scores plots.

We analyzed four hundred fifty-eight lipid species in total in the samples, belonging to twenty-five lipid classes. Eighteen lipids were considered to be different between the two groups according to our criteria (Table 2 and Figure S2). Two-dimensional PCA was applied to develop an overview of the plasma lipidome of patients with CAD and control patients with high plasma HDL-C levels. Data from the LC-MS/MS analysis were pareto-scaled and subject to PCA. As the scores plot of the PCA illustrates in Figure S1, data points for lipids did not show a class separation in an unsupervised manner. Furthermore, we performed PLS-DA to identify the difference in plasma lipidome between the two groups. Although some areas overlapped, the scores plot of two-dimensional PLS-DA demonstrated a dissociation tendency (Figure 1A). In the scores plot of the three-predictive component PLS-DA (Figure 1B), the High HDL CAD (+) group was separated from the High HDL CAD (+) group. The parameters for the explained variation of the PLS-DA model (R2) and predictive values (Q2) were 0.91 and 0.327, respectively. The scores plot described 32.8% of the total variance, including component 1 (8.9%), component 2 (15.8%), and component 3 (8.1%).

### 3.3. Identification and Hierarchical Clustering Analysis of Differential Lipid Species

**Figure 2:**
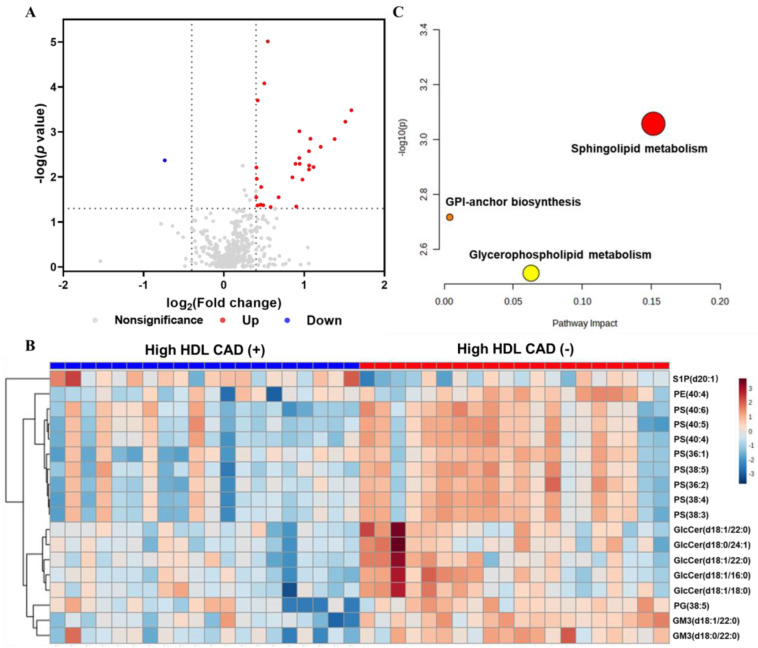
(**A**) Volcano plot of the statistically significant lipids, derived from the comparison between CAD (+) and CAD (−) groups, generated by *p* value from Wilcoxon test and fold change (FC). Red plots indicate lipids are up−regulated in High HDL CAD (+) group and blue means down−regulated. Gray plots represent insignificantly altered lipids. (**B**) Heatmap visualization of significantly altered lipid species between the CAD (+) and CAD (−) groups. (**C**) Pathway analysis of differential lipids between the CAD (+) and CAD (−) groups. GPI= glycosylphosphatidylinositol; S1P = sphingosine−1−phosphate; PE = phosphatidylethanolamine; PS = phosphatidylserine; GlcCer = glucosylceramide; PG = phosphatidylglycerol; GM3 = monosialo−dihexosyl ganglioside.

We found that profiles for lipid metabolites held the most significant discriminating power between groups by VIP scores. Further comparison between the two groups was performed by univariate analyses. We performed a Wilcoxon test and found thirty-four lipid species with statistically significant (*p* < 0.05) differences in levels between the groups (Table S2). After adjusting for age, sex, smoking status, and hs-CRP, there were twenty-two lipids with remarkable *p* values. The FC indicated that one hundred eighteen lipids were down-regulated in the CAD (+) group, whereas three hundred twenty-seven lipids were up-regulated (Figure 2A). The differentially expressed lipid species were identified based on the following criteria: (1) VIP > 1.8, (2) absolute value of log_2_ (FC) > 0.4 (FC > 1.32 or < 0.76), and (3) adjusted *p* < 0.05. A total of eighteen differentially expressed lipid species were confirmed, corresponding to ten glycerophospholipids and eight sphingolipids (Table 2). Except for sphingosine-1-phosphate (S1P) (d20:1), levels for the other seventeen lipids were significantly higher in samples from the High HDL CAD (+) group.

HCA was performed on all of these differential metabolites. The heatmap with a two-dimensional hierarchical clustering revealed a difference between the two groups (Figure 2B). The clustering of species consisted of two principal groups: the first cluster had a single S1P species, S1P (d20:1), which was the only down-regulated differential lipid species in the CAD (+) group, whereas the second cluster had seventeen lipids that were more abundant in the High HDL CAD (+) group than the CAD (−) group. The seventeen lipids included one phosphatidylglycerol (PG), two monosialo-dihexosyl gangliosides (GM3), five glucosylceramides (GlcCer), eight phosphatidylserines (PS), and one phosphatidylethanolamine (PE).

### 3.4. Differential Lipid Metabolite Pathway and Correlation Analysis

**Figure 3:**
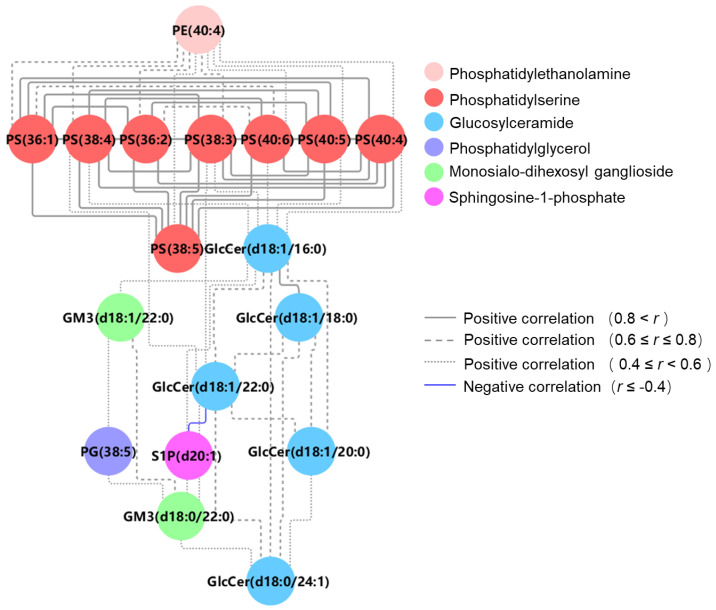
Differential lipid correlation network using the Spearman correlation coefficients. PE = phosphatidylethanolamine; PS = phosphatidylserine; PG = phosphatidylglycerol; S1P = sphingosine−1−phosphate; GlcCer = glucosylceramide; GM3 = monosialo−dihexosyl ganglioside.

To understand how changes in lipid species might influence body metabolism, we carried out a pathway analysis to identify specific metabolic pathways, using enrichment and topology analyses [29]. As shown, we find that sphingolipid and glycerophospholipid metabolism pathways were significantly disrupted, with raw *p* < 0.05 and higher impact (Figure 2C), which may be associated with CAD occurrence in populations with high HDL-C levels. In addition, we performed Spearman’s analysis to assess the correlation between the differential lipid species and calculated coefficients. The correlation network was based on selected pairs with absolute coefficients ≥ 0.4 (Figure 3). As shown, we found that glycerophospholipids were positively correlated with their lipid class and sphingolipids. There, we found that GM3s and GlcCers had a positive relationship, whereas only S1P (d20:1) had a negative correlation with GlcCer (d18:1/18:0). These findings are in accordance with our results with HCA results, implying that S1P (d20:1) may play a complementary role to other lipid species whose levels are significantly different. Additionally, our analysis revealed that several differential lipid species were weakly correlated to lipid data (Table S3). All PSs and several GlcCers were positively related to ApoB, whereas S1P was negatively associated with ApoB (*p* < 0.05). Moreover, several GlcCers had an inverse correlation with HDL.

### 3.5. ROC Analysis and Lipid Biomarker Selection

**Figure 4:**
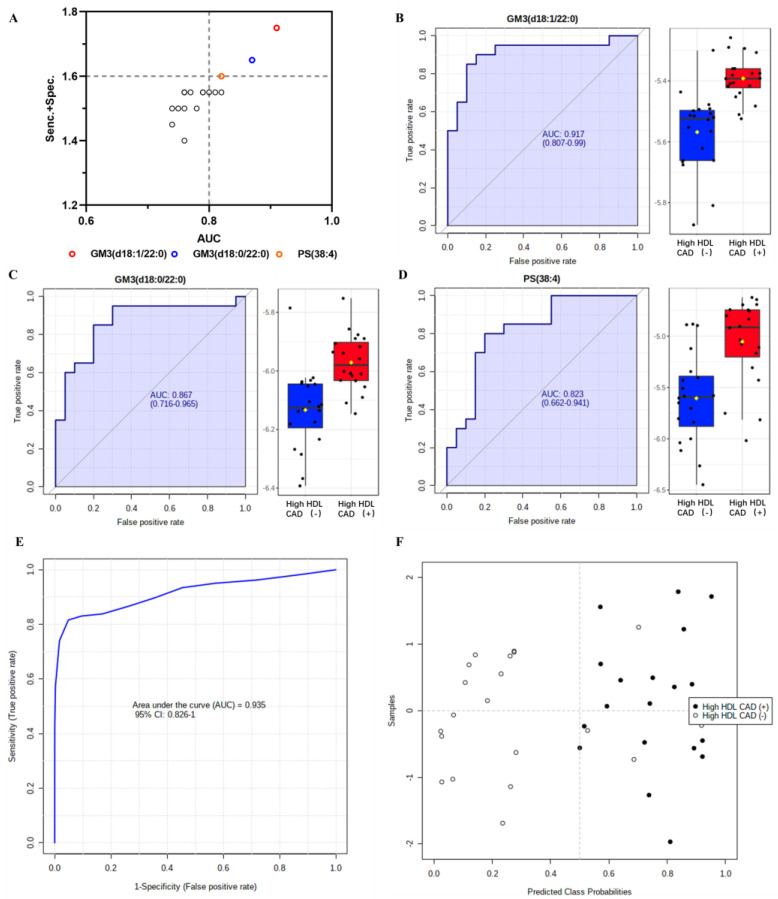
Selection and evaluation of lipid biomarker candidates of CAD with elevated HDL−C levels. (**A**) Screening potential lipid biomarkers using the AUC (*x*−axis) and value of specificity + sensitivity (*y*−axis) calculated by the ROC analysis. Three colored circles represent that AUC of differential lipids are greater than 0.8, and the sum of specificity and sensitivity are greater than 1.6 meanwhile. The white circles represent lipids with the sum of specificity and sensitivity less than 1.6. (**B**−**D**) ROC curves and histograms of GM3 (d18:1/22:0), GM3 (d18:0/22:0), and PS (38:4). (**E**) The ROC curve of the biomarker model generated by combining the three lipid biomarkers based on linear SVM. (**F**) The mean predicted class probabilities for individual samples across the one hundred cross−validations of the diagnostic model. GM3 = monosialo−dihexosyl ganglioside; PS = phosphatidylserine; AUC = area under the curve; Sens. = sensitivity; Spec. = specificity.

We next performed a ROC curve analysis to estimate the diagnostic value of differential lipid metabolites independently, as well as to screen for biomarkers. As shown in Table 2, we found that five of the eighteen differentially expressed lipids had AUC > 0.8 in the univariate ROC analysis (Table 2). In addition to AUC, we considered the sum of sensitivity and specificity. When we analyzed this, we detected three potential lipid biomarkers that could be informative as a diagnostic for CAD in a population with elevated HDL-C levels (Figure 4A). The AUCs of GM3 (d18:1/22:0), GM3 (d18:0/22:0), and PS (38:4) were 0.917, 0.870, and 0.823, respectively (Figure 4B–D). Furthermore, we established a biomarker model with an AUC of 0.935 by combining the three identified biomarker candidates (Figure 4E,F). Next, we used a linear support vector machine (SVM) as a classification method with one hundred cross-validations, and we showed a greater diagnostic potency than with individual lipid candidates (Figure 4F). Additionally, the average of the predicted class probabilities of the model could accurately distinguish nineteen samples from the twenty patients with CAD, and could discriminate sixteen individuals from the twenty controls (Table S4).

## 4. Discussion

High HDL-C levels have been documented to have an inverse association with CAD. However, patients with CAD can have high HDL-C levels, which presents as a potential confound. As such, the underlying mechanism of CAD incidence in individuals with elevated HDL-C remains to be better understood. Here, we have explored the changes in lipid composition in the context of high HDL-C in CAD using lipidomic methods to address this issue. We compared four hundred fifty-eight lipid species obtained from a study cohort comprising patients with CAD and a control group without CAD; however, all of these patients had elevated HDL levels, because we reasoned that this model would help us understand how changes to the lipidome might be relevant to CAD in patients with elevated HDL-C levels. To our knowledge, this is the first study to explore the plasma lipidome of individuals with high HDL-C levels.

Our study has led to the finding that levels of eighteen plasma lipid species are significantly different between patients with CAD, and those without CAD. To reduce the impact of drugs and comorbidities on lipidome, patients using statins within the past three months before enrollment and those complicated with specific diseases were excluded, since other medications affecting lipids are not commonly prescribed in clinical practice. In addition, age and smoking status influence the concentrations and compositions of HDL. Thus, we identified the differential lipids according to *p* values adjusted by age, smoking status, and other potential confounding variables. Among differential lipid species, levels of seven sphingolipids and ten glycerophospholipids were detected to be higher in patients with CAD, while the level of one sphingolipid was lower in the CAD group. These CAD-related sphingolipids corresponded to three subclasses of lipids: GlcCer, S1P, and GM3. In contrast, the altered glycerophospholipids in the CAD group belonged to three categories of lipids: PS, PG, and PE. Further clustering analysis drew the same conclusion. Considering our findings more broadly, our results on the differential lipids detected in patients with CAD and high HDL levels stand in contrast with findings from earlier studies. For example, previous studies of the plasma lipidome between CAD patients and healthy controls showed alterations in levels of Cers, Pes, phosphatidylinositols, phosphatidylcholines (PC), lyso-PCs, and lyso-PEs [16,30]. Specific to those previous reports, changes in the levels of Cer (d18:1/16:0), Cer (d18:1/18:0), and Cer (d18:1/24:1) were documented in patients with CAD [30,31]; however, we did not find significant differences in these lipid species in our study. Altogether, these findings could suggest that lipid species associated with CAD may be particular to individuals with high HDL-C levels.

Among the glycerophospholipids related to CAD, levels of eight PSs were found to be increased in CAD patients with high HDL levels. As a member of the cell membrane phospholipids, PS plays an important role in cell apoptosis and blood clotting [32]. PS is ordinarily asymmetrically localized to the internal layer of the plasma membrane. If activated or injured, cells expose PS to the external leaflet of their plasmalemma and form a pattern that is subsequently recognized by macrophages and antibodies [33]. PS externalization of cardiomyocytes occurs after a short ischemic insult in a rabbit model and is associated with apoptosis [34]. Accumulation of PS has been detected in atherosclerotic plaques [35], and some PSs increase in patients with atherosclerosis [36]. The elevated PSs in patients with CAD in our study may indicate that increased cell damage and apoptosis occur in patients with CAD.

Another major dysregulated glycerophospholipid subclass identified between the two groups is GlcCers. Our results showed that the levels of five GlcCers were higher in patients with CAD and high HDL-C levels compared to controls. Currently, studies on the role of GlcCer have reported different conclusions. Some studies showed that GlcCer has an anticoagulant effect and may protect the cardiovascular system [37], and inhibiting mouse GlcCer synthase can reduce atherosclerosis [38]. Additionally, GlcCers have been observed to accumulate in atherosclerotic plaques [39], and GlcCers promote plaque inflammation and vascular smooth muscle cell apoptosis in the formation of atherosclerosis [40]. Combined with previous findings, we conclude that GlcCer may have proatherogenic effects on individuals with elevated HDL-C levels.

Of the sphingolipids identified, we found that S1P (d20:1) was the only lipid downregulated in the CAD group and negatively correlated with other altered lipid metabolites. It is known that S1P is mainly carried by HDL in plasma and has a double effect on the pathogenesis of CAD [41]. Although some studies suggest that HDL-bound S1P has atherosclerotic protective properties by promoting certain functions of HDL, such as anti-inflammation and vasodilation [42], free S1P has been shown to have a pro-inflammatory effect and to be harmful to cells of the vascular system [43]. In this study, plasma S1P species were measured without separately studying HDL-bound S1P and free S1P. Consistent with previous results [43], our findings revealed a decrease in plasma S1P (d20:1) levels in patients with CAD and high HDL-C levels. More importantly, we found no significant difference in high HDL-C levels existed between the CAD and non-CAD groups, which suggests that the decrease in S1P (d20:1) may be an independent protective factor for patients with CAD and elevated HDL-C levels.

We determined that sphingolipid and glycerophospholipid metabolic pathways are the most closely related to the pathogenesis of CAD, and these findings are consistent with previous studies [44,45]. Aberrant sphingolipid metabolism is implicated in the progression of atherosclerosis [45] and plaque inflammation [40]. Reduced sphingolipid metabolism has been found to attenuate cell death and inflammation after myocardial infarction [18]. Previous studies have suggested that the total plasma phospholipids of patients with CAD and high HDL-C levels are lower than those of the control group, and different phospholipids have not been analyzed [22]. Our study is consistent with these previous studies, and we propose that the increase in glycerophospholipid levels may be related to CAD. Additionally, glycerophospholipids have been reported as potential immune-inflammatory mediators in CAD [18,19]. Indeed, glycerophospholipid metabolism is one of the most severely disturbed pathways in the progression of atherosclerosis [45], and our results are consistent with this. Hence, we hypothesize that even if the HDL-C level is favorable, dysregulation of sphingolipid metabolism as well as glycerophospholipid metabolism may serve to aggravate plaque progression through inflammation and other mechanisms, leading to the onset of CAD.

The potential of plasma differential lipids as a prospective diagnostic biomarker for CAD in a population with elevated HDL-C levels was examined in this study. Three lipid species, GM3 (d18:1/22:0), GM3 (d18:0/22:0), and PS (38:4) were informative as discriminatory markers compared with other lipid species, all of which were higher in patients with CAD. Moreover, the model amalgamating the three selected lipids had greater statistical power to differentiate between the two groups. Emerging studies have explored the value of lipid biomarkers in CAD diagnosis and prognosis prediction [46,47]. Machine learning revealed that alterations to serum sphingolipids are informative as biomarkers of CAD and can be used to improve risk stratification [47]. Nevertheless, biomarkers of prognostic value for CAD with high HDL-C concentrations remain to be validated. Our study suggests that alterations to the HDL lipidome may be informative as a biomarker to distinguish individuals with residual risk of CAD, even when HDL-C is elevated in these patients [21]. Our findings remain to be validated through larger population studies.

We find that our study had several limitations. First, our study was exploratory work in a single hospital with a limited sample size due to strict inclusion criteria (we enrolled patients with high HDL-C levels without a history of diabetes mellitus and statin use). The small sample size of the study reduced the likelihood of observing statistically significant results in analysis, thus covering the true discrimination of lipidomics between the two groups. Our findings should be validated independently in a multi-center study with a larger sample size of patients with comorbidities. This study should also include participants recruited from communities. Second, since the study was an observational cross-sectional study, a causal relationship between the dysregulated plasma lipidome and CAD could not be established. To establish causality, independent follow-up, and interventional studies should be conducted on the candidate lipids identified. Third, lipoprotein lipidomics studies were carried out, which could have been more comprehensive. As such, while our studies have led to the identification of putative markers for validation in large-scale replication studies, our understanding of the precise mechanisms for lipid dysregulation in CAD remains to be better explored since the subclasses, sizes and lipidome of HDL were not evaluated. Further research to explore plasma and lipoprotein lipidomics, and other biological and immunological characteristics in CAD patients with high HDL-C levels will be needed to elucidate the exact mechanism of lipid dysregulation for this condition. The subclasses and sizes of HDL should also be detected in future studies. Fourth, lifestyle factors and nutritional habits, which might influence concentrations of HDL-C, were not available in our data. Studies including this information are essential to comprehensively understand the role of lipidome in CAD occurrence in the context of high HDL-C levels. Finally, the cost of lipidome detection restricts its clinical use. Compared with lipidome, lipid biomarkers could be helpful to clinical practice after being confirmed by extensive studies. Similar studies with a selection of crucial lipids are essential to reduce costs and wide usage.

## 5. Conclusions

Our study is the first to investigate the plasma lipidome of CAD in individuals with favorable HDL-C levels. We found that lipidomic profiles were significantly different between patients with CAD and controls with elevated HDL-C levels. Differentially expressed lipids were identified by multivariate and univariate analyses, which corresponded largely to PS and GlcCer species. Such differences may be relevant to CAD through a mechanism involving disruptions to sphingolipid and glycerophospholipid metabolism. Furthermore, we have identified several putative lipid biomarkers as diagnostic indicators for CAD, and these have the potential for validation as prognostic markers for this condition. Our findings indicate that pathological lipid metabolism underlies CAD.

## Figures and Tables

**Table 1:** Summary of clinical and demographic characteristics of enrolled patients.

Characteristics	High HDL CAD (−) (*n* = 20)	High HDL CAD (+) (*n* = 20)	*p* Value
Age (years)	59 (54, 64)	62 (59, 66)	0.086
Male (%)	50	65	0.523
Waist circumference (cm)	87.10 ± 8.74	89.82 ± 9.97	0.364
BMI (kg/m^2^)	22.97 ± 2.60	23.95 ± 4.03	0.372
Smokers (%)	40	70	0.055
Hypertension history (%)	50	50	1.000
HDL-C (mmol/L)	1.51 (1.40, 1.69)	1.53 (1.32, 1.58)	0.277
TC (mmol/L)	4.84 ± 0.78	5.18 ± 1.06	0.253
LDL-C (mmol/L)	2.60 ± 0.75	2.99 ± 0.88	0.151
TG (mmol/L)	1.06 (0.79, 1.34)	1.23 (0.82,1.85)	0.253
Apo A1 (mg/L)	1979.80 ± 293.12	1929.35 ± 307.83	0.599
Apo B (mg/L)	765.90 ± 233.79	942.35 ± 271.61	0.034 ^a^
Lp(a) (mg/L)	90.00 (41.00, 247.75)	231.00 (72.25, 422.00)	0.046 ^a^
Fasting glucose(mmol/L)	5.11 ± 0.65	5.13 ± 0.84	0.933
HbA1C (%)	5.49 ± 0.33	5.67 ± 0.41	0.123
hs-CRP (mg/L)	8.73 ± 6.72	5.07 ± 3.61	0.040 ^a^

^a^ *p* < 0.05. Continuous variables are expressed as the means ± standard deviation or median (interquartile range). Dichotomous variables are expressed as %. BMI = body mass index; HDL-C = high-density lipoprotein cholesterol; TC = total cholesterol; LDL-C = low-density lipoprotein cholesterol; TG = total triglyceride; Apo A1 = apolipoprotein A1; Apo B = apolipoprotein B; Lp (a) = lipoprotein (a); HbA1C = hemoglobin A1C; hs-CRP = hypersensitive C-reactive protein.

**Table 2:** Statistical analysis of plasma differential lipid species to distinguish the High HDL CAD (+) group from the High HDL CAD (−) group.

Lipid Species	Adjusted *p* Value ^a^	VIP ^b^	FC ^c^	AUC (95% CI) ^d^	Sensitivity ^d^	Specificity ^d^	Sens. + Spec.
Sphingolipid							
GM3 (d18:1/22:0)	0.010	2.57	1.46	0.917 (0.807–0.990)	0.90	0.85	1.75
GM3 (d18:0/22:0)	0.009	2.30	1.42	0.870 (0.730–0.964)	0.85	0.80	1.65
GlcCer (d18:1/16:0)	0.026	2.78	2.08	0.779 (0.621–0.914)	0.80	0.70	1.50
GlcCer (d18:0/24:1)	0.015	2.22	1.85	0.765 (0.591–0.899	0.80	0.75	1.55
GlcCer (d18:1/22:0)	0.037	2.39	2.08	0.751 (0.586–0.900)	0.85	0.65	1.50
GlcCer (d18:1/18:0)	0.034	2.33	1.81	0.739 (0.571–0.889)	0.90	0.60	1.50
GlcCer (d18:1/20:0)	0.020	2.27	1.97	0.735 (0.555–0.866)	0.95	0.50	1.45
S1P (d20:1)	0.015	2.34	0.60	0.760 (0.615–0.889)	0.75	0.65	1.40
Glycerophospholipid							
PS (38:4)	0.008	4.16	3.01	0.823 (0.662–0.941)	0.80	0.80	1.60
PS (38:3)	0.008	3.97	2.85	0.818 (0.666–0.944)	0.85	0.70	1.55
PS (36:1)	0.012	3.04	1.92	0.809 (0.661–0.925)	0.85	0.70	1.55
PS (36:2)	0.014	3.05	2.11	0.796 (0.666–0.909)	0.75	0.80	1.55
PS (40:4)	0.010	3.72	2.60	0.792 (0.636–0.929)	0.80	0.75	1.55
PS (38:5)	0.034	3.42	2.31	0.785 (0.645–0.922)	0.75	0.80	1.55
PS (40:6)	0.010	3.33	2.09	0.758 (0.579–0.887)	0.70	0.85	1.55
PS (40:5)	0.019	3.00	2.17	0.755 (0.560–0.906)	0.70	0.85	1.55
PE (40:4)	0.024	2.82	1.92	0.758 (0.599–0.891)	0.90	0.60	1.50
PG (38:5)	0.037	3.49	1.92	0.769 (0.604–0.899)	0.65	0.90	1.55

^a^ *p* value was adjusted by age, sex, smoking status, and hs-CRP with a threshold of 0.05. ^b^ VIP was obtained from PLS-DA model with a threshold of >1.8. ^c^ FC was calculated based on mean ratios for lipids of patients with CAD to lipids of controls. ^d^ AUC, sensitivity, and specificity were obtained from ROC analysis. VIP = variable importance in projection; FC = fold change; AUC = area under the curve; CI = confidence interval; Sens. = sensitivity; Spec. = specificity; GM3 = monosialo-dihexosyl ganglioside; GlcCer = glucosylceramide; S1P = sphingosine-1-phosphate; PS = phosphatidylserine; PE = phosphatidylethanolamine; PG = phosphatidylglycerol.

## Data Availability

The data presented in this study are available on request from the corresponding author. The data are not publicly available due to ethical concerns.

## Supplementary Materials

The following supporting information can be downloaded at: https://www.mdpi.com/article/10.3390/metabo13060695/s1, Figure S1: (A) Principal component analysis (PCA) scores plot of CAD High HDL and Control High HDL with 95% confidence regions. (B) PCA scree plot; Figure S2: Boxplots of differential lipid species; Figure S3. Volcano plot of the statistically significant lipids based on normalized data. Table S1: Resources of materials; Table S2: Lipid species with statistical significance by Wilcoxon test; Table S3: Differential lipid species to distinguish the High HDL CAD (+) group from the High HDL CAD (−) group based on normalized data. Table S4: Spearman correlation coefficients between differential lipid species and lipid data; Table S5: Confusion matrix of predicted class probabilities across the 100 cross-validations.
