# Supplementary material for: Coronary Artery Disease with Elevated Levels of HDL Cholesterol Is Associated with Distinct Lipid Signatures
Source: Metabolites. 2023 May 26;13(6):695. doi: 10.3390/metabo13060695 (PMC10301469; doi:10.3390/metabo13060695)
Supplement: Supplementary file 1 [file metabolites-13-00695-s001.zip › metabolites-2372115-supplementary.pdf]

Supplementary materials

Original article

Coronary Artery Disease with Elevated Levels of HDL Cholesterol is Associated with Distinct Lipid Signatures

Wanying Xia[a], Haiyi Yu[a *], Guisong Wang[a *]

[a] Department of Cardiology and Institute of Vascular Medicine, Peking University Third Hospital; NHC Key Laboratory of Cardiovascular Molecular Biology and Regulatory Peptides; Key Laboratory of Molecular Cardiovascular Science, Ministry of Education; Beijing Key Laboratory of Cardiovascular Receptors Research, No. 49 North Garden Road, Haidian District, Beijing 100191, China

*Corresponding author:

Haiyi Yu, PhD

Department of Cardiology and Institute of Vascular Medicine, Peking University Third Hospital, No. 49 North Garden Road, Haidian District, Beijing 100191, China

Email: yuhaiyi@bjmu.edu.cn

Guisong Wang, MD

Department of Cardiology and Institute of Vascular Medicine, Peking University Third Hospital, No. 49 North Garden Road, Haidian District, Beijing 100191, China

Phone: +86015611908831

Fax: 86-10-62361450

Email: guisongwang@bjmu.edu.cn

Section 1: Resources of materials

Table S1. Resources of materials.

Material	Manufacturer	Identifier
Chloroform (HPLC grade)	Honeywell	049-4
Methanol (HPLC grade)	Fisher chemical	A452-4
Luna 3 μm-silica column	Phenomenex	00F-4162-b0
Kinetex-C18 2.6 mm column	Phenomenex	00D-4462-e0
PE-14:0/14:0	Avanti Polar Lipids	850745P
d_{31}-PE-(16:0/18:1)	Avanti Polar Lipids	860374C
d_{31}-PS-(16:0/18:1)	Avanti Polar Lipids	860403C
PA-17:0/17:0	Avanti Polar Lipids	830856P
PG-14:0/14:0	Avanti Polar Lipids	840445P
d_{31}-PG- (16:0/18:1)	Avanti Polar Lipids	860384C
d_{31}-PI-(16:0/18:1)	Avanti Polar Lipids	860042P
SM-d18:1/12:0	Avanti Polar Lipids	860583P
LPC-17:0	Avanti Polar Lipids	855676P
LPE-17:1	Avanti Polar Lipids	110699
LPI-17:1	Avanti Polar Lipids	850103P
LPA-17:0	Avanti Polar Lipids	857127P
LPS-17:1	Avanti Polar Lipids	858141P
S1P-d17:1	Avanti Polar Lipids	860641P
Cer-d18:1/17:0	Avanti Polar Lipids	860517P
GlcCer-d18:1/8:0	Avanti Polar Lipids	860540P
GalCer-d18:1/8:0	Avanti Polar Lipids	860538P
PI-8:0/8:0	Echelon	P-0008
d_3-GM3-d18:1/18:0	Matreya LLC	2052
d_3-LacCer-d18:1/16:0	Matreya LLC	1534
d_5-DAG-16:0/16:0	Avanti Polar Lipids	110537

d_5-DAG-18:1/18:1	Avanti Polar Lipids	110581
d_5-TAG-(14:0)3	C/D/N Isotopes	D-6958
d_5-TAG-(16:0)3	C/D/N Isotopes	D-5815
d_5-TAG-(18:0)3	C/D/N Isotopes	D-5816
d_6-cholesterol	C/D/N Isotopes	D-2139
d_6-CE18:0	C/D/N Isotopes	D-5823

PE = phosphatidylethanolamine; PS = phosphatidylserine; PA = phosphatidic acid; PG = phosphatidylglycerol; PI phosphatidylinositol; SM = sphingomyelin; LPC = lyso-phosphatidylcholine; LPE = lyso-PE; LPI = lyso-PI; LPA = lyso-PA; LPS = lyso-PS; S1P = sphingosine-1-phosphate; Cer = ceramide; GlcCer = glucosylceramide; Galcer = galactosylceramide; GM3 = monosialo-dihexosyl ganglioside; LacCer = lactosylceramide; DAG = diacylglycerol; TAG = triacylglycerol; CE = cholesteryl ester.

Section 2: Supplementary results

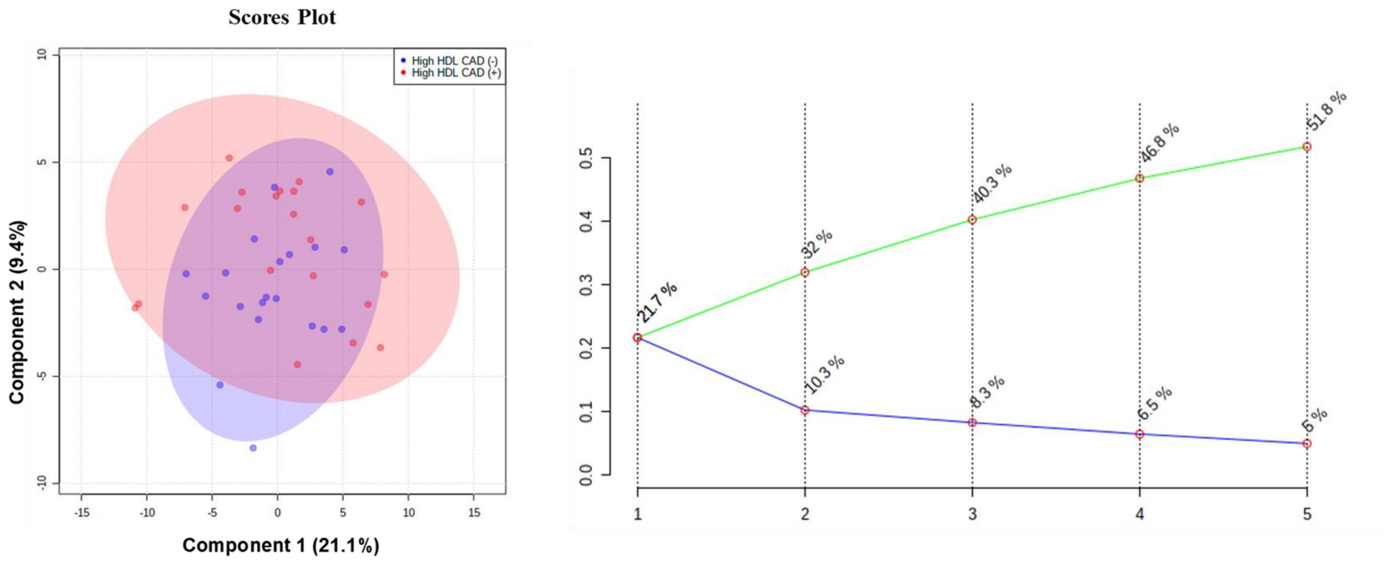

Figure S1. (A) Principal component analysis (PCA) scores plot of CAD High HDL and Control High HDL with 95% confidence regions (each sample is represented by a point). (B) PCA scree plot (the green line on top shows the accumulated variance explained; the blue line underneath shows the variance explained by individual principal components).

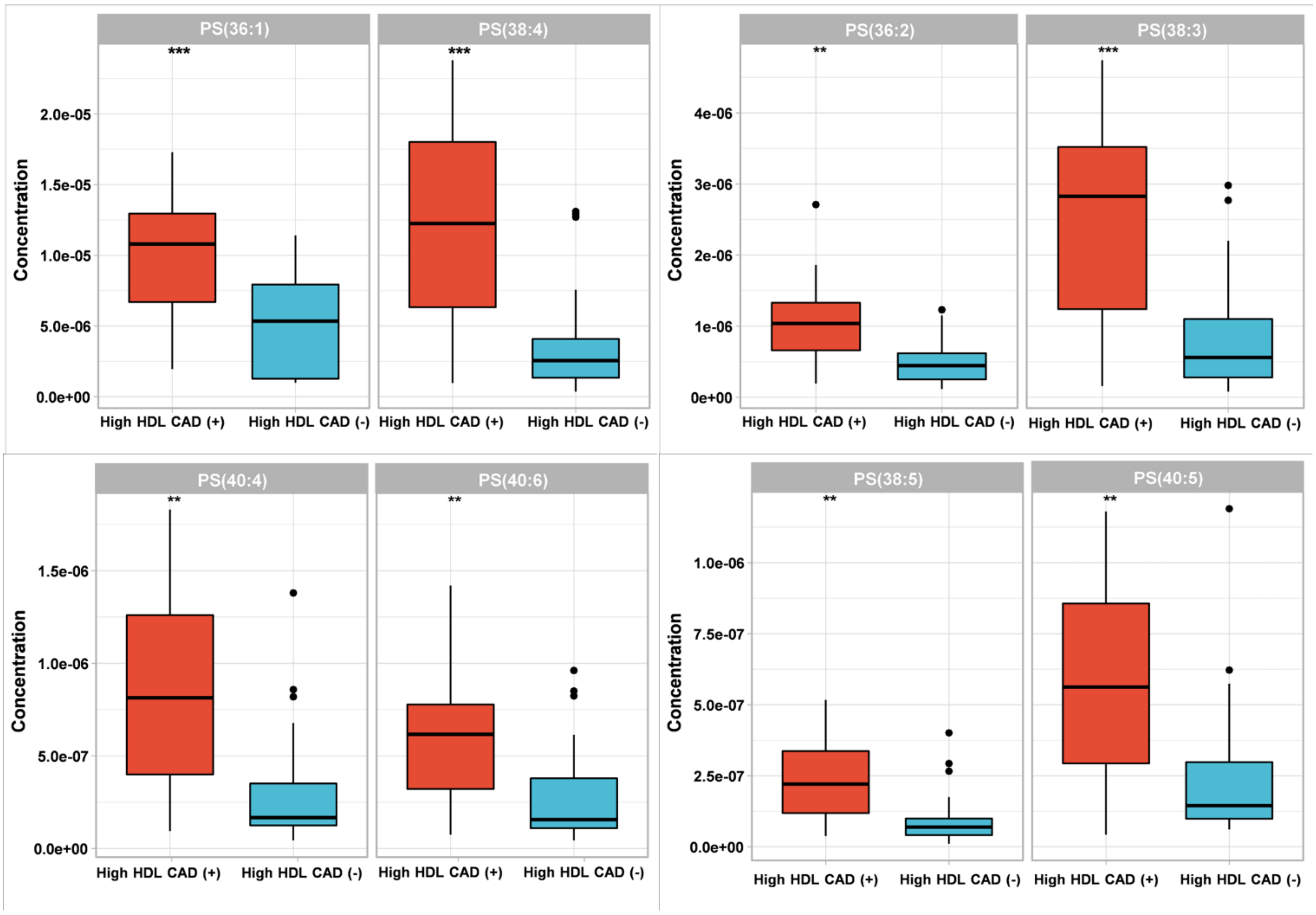

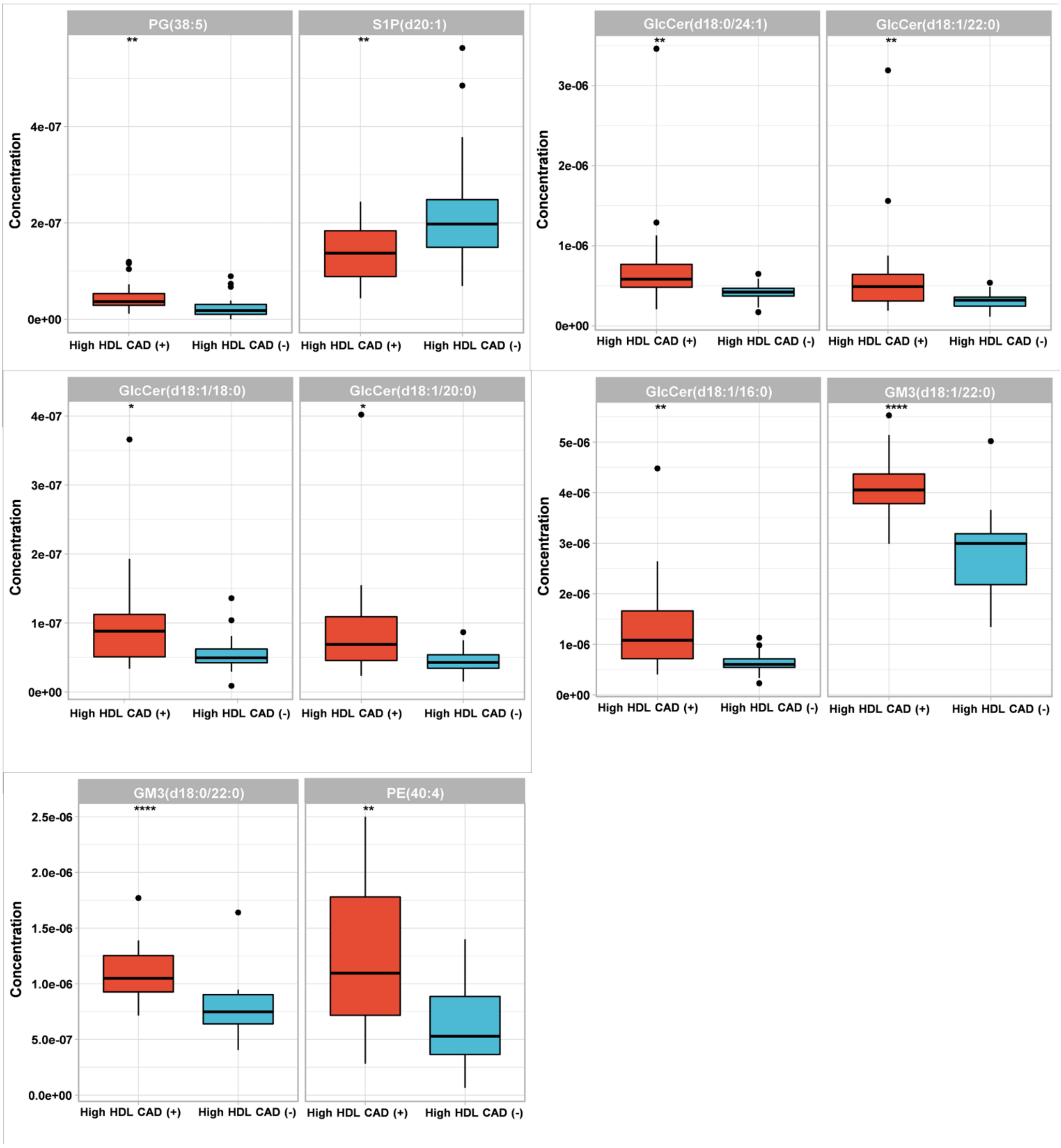

Figure S2. Boxplots of differential lipid species.
PE = phosphatidylethanolamine; PS = phosphatidylserine; PG = phosphatidylglycerol; S1P = sphingosine–1–phosphate; GlcCer = glucosylceramide; GM3 = monosialo–dihexosyl ganglioside.

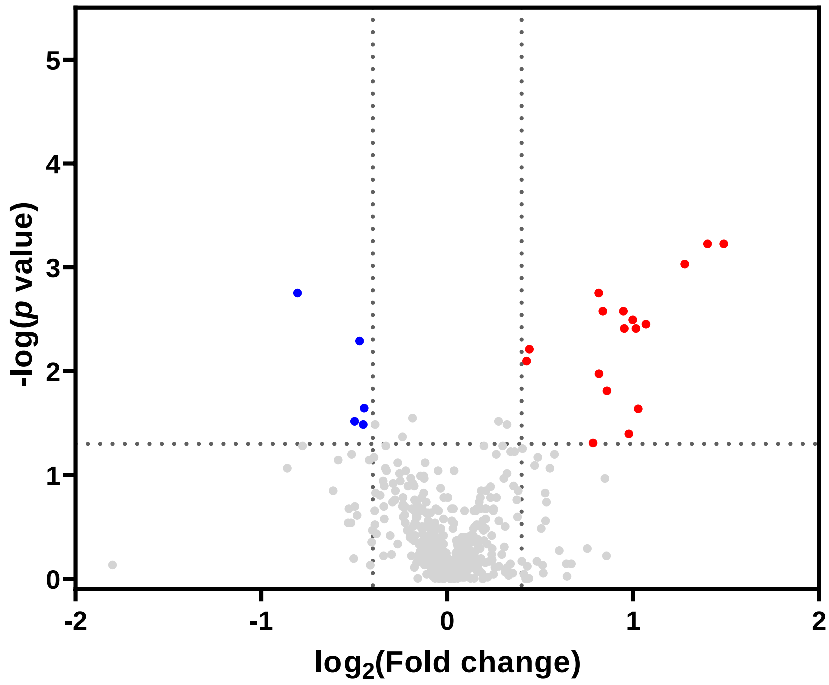

Figure S3. Volcano plot of the differential lipids based on normalized data.
Red plots indicate lipids are up−regulated in High HDL CAD (+) group and blue means down−regulated. Gray plots represent insignificantly altered lipids.

Table S2. Lipid species with statistical significance by Wilcoxon test.

Lipid species	*p* value	Adjusted *p* value	Lipid species	*p* value	Adjusted *p* value
CE(18:2)	0.006	0.033	PS(40:5)	< 0.001	0.019
PA(36:1)	0.033	0.047	PS(40:6)	0.002	0.010
PE(34:2)	0.045	0.071	LPE(18:0)	0.001	0.482
PE(36:1)	0.020	0.063	LPS(18:1)	0.006	0.146
PE(38:3)	0.006	0.121	LPC(18:2)	0.006	0.037
PE(38:4)	0.041	0.111	TAG42:0(14:0)	0.043	0.149
PE(38:6p)	0.021	0.107	TAG44:1(14:1)	0.042	0.053
PE(40:4)	0.017	0.024	Cer(d18:1/24:0)	0.028	0.220
PE(42:5)	0.011	0.170	GlcCer(d18:0/24:1)	0.005	0.015
PG(38:5)	0.026	0.037	GlcCer(d18:1/16:0)	0.003	0.026
PG(38:6)	0.005	0.080	GlcCer(d18:1/18:0)	0.010	0.034

PS(36:1)	0.028	0.012	GlcCer(d18:1/20:0)	0.011	0.020
PS(36:2)	0.004	0.014	GlcCer(d18:1/22:0)	0.007	0.037
PS(38:3)	0.046	0.008	GM3(d18:0/22:0)	< 0.001	0.009
PS(38:4)	0.001	0.008	GM3(d18:1/22:0)	< 0.001	0.010
PS(38:5)	0.001	0.034	GM3(d18:1/24:0)	< 0.001	0.009
PS(40:4)	0.001	0.010	S1P(d20:1)	0.004	0.015

CE = cholesteryl ester; PA = phosphatidic acid; PE = phosphatidylethanolamine; PG = phosphatidylglycerol; PS = phosphatidylserine; LPE = lyso-PE; LPS = lyso-PS; LPC = lyso-phosphatidylcholine; TAG = triacylglycerol; Cer = ceramide; GlcCer = glucosylceramide; GM3 = monosialo-dihexosyl ganglioside; S1P = sphingosine-1-phosphate.

Table S3. Differential lipid species to distinguish the High HDL CAD (+) group from the High HDL CAD (-) group based on normalized data.

Lipid species	*p* value [a]	Adjusted *p* value [b]	FC [c]	VIP [d]
Sphingolipid				
GM3(d18:1/22:0)	0.023	0.010	2.039	2.05
GM3(d18:0/22:0)	0.008	0.009	1.344	1.97
GlcCer(d18:1/16:0)	0.003	0.026	1.997	2.65
GlcCer(d18:0/24:1)	0.015	0.015	1.814	2.00
GlcCer(d18:1/22:0)	0.023	0.037	2.039	2.22
GlcCer(d18:1/18:0)	0.049	0.034	1.723	2.16
GlcCer(d18:1/20:0)	0.023	0.020	2.039	2.06
S1P(d20:1)	0.002	0.015	0.573	2.79
Glycerophospholipid				
PS(38:4)	0.001	0.008	2.803	4.35
PS(38:3)	0.001	0.008	2.641	4.17
PS(36:1)	0.002	0.012	1.760	3.05
PS(36:2)	0.003	0.014	1.928	3.07
PS(40:4)	0.011	0.010	1.761	3.86
PS(38:5)	0.004	0.034	2.098	3.49
PS(40:6)	0.004	0.010	1.934	3.36
PS(40:5)	0.004	0.019	2.021	3.05
PE(40:4)	0.001	0.024	2.425	2.79
PG(38:5)	0.004	0.037	2.098	3.62
PC(36:4p)	0.005	0.084	0.721	2.02
Glycerolipid				
TAG51:3(15:0)	0.023	0.221	0.734	1.84

[a] *p* value generated by Wilcoxon test with a threshold of 0.05.

[b] *p* value was adjusted by age, sex, smoking status, and hs-CRP.

[c] VIP was obtained from PLS-DA model with a threshold of > 1.8.

[d] FC was calculated based on mean ratios for lipids of patients with CAD to lipids of controls.

VIP = variable importance in projection; FC = fold change; GM3 = monosialo-dihexosyl ganglioside; GlcCer = glucosylceramide; S1P = sphingosine-1-phosphate; PS = phosphatidylserine; PE = phosphatidylethanolamine; PG = phosphatidylglycerol; PC = psphatidylcholine; TAG = tiacylglycerol.

Table S4. Spearman correlation coefficients between differential lipid species and lipid data.

	TC	LDL	HDL	TG	ApoA1	ApoB	Lp(a)
PE(40:4)	0.224	0.181	-0.161	0.262	0.041	0.3	-0.034
PS(36:2)	0.323[a]	0.357[a]	-0.195	0.263	0.005	0.416[a]	0.146
PS(36:1)	0.303	0.334[a]	-0.185	0.25	0.022	0.409[a]	0.142
PS(38:5)	0.364[a]	0.414[a]	-0.224	0.354[a]	0.019	0.487[a]	0.222
PS(38:4)	0.332[a]	0.364[a]	-0.162	0.282	0.057	0.452[a]	0.2
PS(38:3)	0.330[a]	0.370[a]	-0.202	0.284	0.005	0.441[a]	0.218
PS(40:6)	0.324[a]	0.348[a]	-0.372[a]	0.434[a]	-0.017	0.471[a]	0.311
PS(40:5)	0.415[a]	0.424[a]	-0.318[a]	0.428[a]	0.045	0.530[a]	0.209
PS(40:4)	0.354[a]	0.383[a]	-0.321[a]	0.372[a]	-0.008	0.483[a]	0.209
PG(38:5)	0.134	0.065	0.003	0.135	0.253	0.094	0.176
GM3(d18:1/22:0)	0.181	0.142	-0.028	0.173	0.041	0.22	0.313[a]
GM3(d18:0/22:0)	0.248	0.259	-0.101	0.126	0.064	0.258	0.232
S1P(d20:1)	-0.179	-0.24	0.098	-0.18	-0.043	-0.406[a]	-0.395[a]
GlcCer(d18:1/16:0)	0.249	0.315[a]	-0.425[a]	0.151	-0.302	0.383[a]	0.353[a]
GlcCer(d18:1/18:0)	0.128	0.186	-0.423[a]	0.212	-0.298	0.331[a]	0.365[a]
GlcCer(d18:1/20:0)	0.171	0.226	-0.356[a]	0.285	-0.186	0.347[a]	0.307
GlcCer(d18:1/22:0)	0.294	0.393[a]	-0.262	0.14	-0.195	0.413[a]	0.245
GlcCer(d18:0/24:1)	0.027	0.106	-0.417[a]	0.116	-0.380[a]	0.161	0.286

[a] $p < 0.05$;

PE = phosphatidylethanolamine; PS = phosphatidylserine; PG = phosphatidylglycerol; S1P = sphingosine-1-phosphate; GM3= monosialo-dihexosyl ganglioside; GlcCer = glucosylceramide; TC = total cholesterol; LDL-C = low density lipoprotein cholesterol; HDL-C = high density lipoprotein cholesterol; TG = total triglyceride; Apo A1 = apolipoprotein A1; Apo B = apolipoprotein B; Lp(a) = lipoprotein(a)

Table S5. Confusion matrix of predicted class probabilities across the 100 cross-validations.

Group	High HDL CAD (+)	High HDL CAD (-)
High HDL CAD (+)	19	4
High HDL CAD (-)	1	16
